# Expression of leukosialin (CD43) defines a major intrahepatic T cell subset associated with protective responses in visceral leishmaniasis

**DOI:** 10.1186/s13071-015-0721-9

**Published:** 2015-02-19

**Authors:** Dirlei Nico, Naiara Maran, Leonardo Santos, Erivan Schnaider Ramos-Junior, Natália Rodrigues Mantuano, Joseane Lima Prado Coutinho, Andre Macedo Vale, Celio Geraldo Freire-de-Lima, Adriane Todeschini, Juliany Cola Fernandes Rodrigues, Clarisa Beatriz Palatnik-de-Sousa, Alexandre Morrot

**Affiliations:** Departamento de Microbiologia Geral, Instituto de Microbiologia, Universidade Federal do Rio de Janeiro, Rio de Janeiro, Brazil; Departamento de Imunologia, Instituto de Microbiologia, Universidade Federal do Rio de Janeiro (UFRJ), CCS - Sala D1-035, Av. Carlos Chagas Filho, 373 - Cidade Universitária, CEP 21.941-902, Ilha do Fundão, Rio de Janeiro, RJ Brazil; Instituto de Biofísica Carlos Chagas Filho, Universidade Federal do Rio de Janeiro, Rio de Janeiro, Brazil

**Keywords:** Visceral leishmaniasis, *Leishmania (L.) infantum chagasi*, Leukosialin (CD43), Intrahepatic T cell subsets, Host protective responses

## Abstract

**Background:**

Leishmaniasis is a neglected vector-borne tropical disease caused by *Leishmania* protozoa that are transmitted to mammalian hosts by infected sand flies. Infection is associated with distinct clinical manifestations that include cutaneous, mucocutaneous and visceral lesions. Visceral leishmaniasis (VL) is the most severe form of the disease and is considered second in terms of mortality and fourth in terms of morbidity among tropical diseases. IFN-γ-producing T cells are involved in protection against the disease.

**Methods:**

CD43^+/+^ and CD43^-/-^ mice on a C57BL/6 background were intravenously injected with 5 × 10 ^7^ amastigotes of *Leishmania (L.) infantum chagasi,* and 30 days after infection the clinical signs of disease were examined; the splenocytes were isolated and assayed for cytokine production; and the livers were removed for phenotypic analysis of T cell subsets by flow cytometry.

**Results:**

We report that mice lacking CD43 display increased susceptibility to infection by *Leishmania (L.) infantum chagasi*, with higher parasite burdens than wild-type mice. The increased susceptibility of CD43^−/−^ mice were associated with a weakened delayed hypersensitivity response and reduced levels of IgG2a antibodies to leishmania antigens. We further showed that expression of CD43 defines a major intrahepatic CD4^+^ and CD8^+^ T cell subsets with pro-inflammatory phenotypes and leads to increased levels of IFN-γ secretion by activated splenocytes.

**Conclusions:**

Our findings point to a role of CD43 in the development of host resistance to visceral leishmaniasis.

**Electronic supplementary material:**

The online version of this article (doi:10.1186/s13071-015-0721-9) contains supplementary material, which is available to authorized users.

## Background

Infection with leishmania spps can induce diseases varying from local cutaneous lesions to systemic visceral manifestations. Parasites of the intracellular protozoan, Leishmania, are transmitted to mammalian hosts by sand fly vectors. The parasites have a dimorphic life cycle consisting of extracellular promastigotes in the vector, and intracellular amastigotes inside mammalian macrophages [1]. The different pathologies are associated with different degrees of parasite spread. Parasite species such as *L. major* are confined to cutaneous lesions while others such as *L. donovani* and *Leishmania (L.) infantum chagasi* have the capability to disseminate into visceral organs such as spleen and liver, and bone marrow causing visceral leishmaniasis, the most severe form of the disease [2]

Adaptive immunity against leishmaniasis is associated with development of T cell-mediated interferon-gamma (IFN-γ) responses. IFN-γ secreted by CD4^+^ and CD8^+^ T cells mediates the respiratory burst in activated macrophages which is responsible for the production of nitric oxide needed for parasite killing inside reservoir cells [3,4]. Other cytokines can modulate the anti-parasite activity promoted by IFN-γ. It has been demonstrated that IL-12 is able to promote Th1 cell-associated mechanisms by inducing IFN-γ, which activates macrophages to kill intracellular parasites in granulomas formed in parasitized tissue foci [5,6]. While it has been shown that TNF-α is able to enhance the macrophage leishmanicidal activity induced by IFN-γ, increased levels of IL-10 oppose macrophage activation by blocking Th1 cellular responses [4,7].

Other host determinants are associated to subclinical and symptomatic infections by leishmania species. In the visceral form of the disease, Nramp1 (natural resistance-associated macrophage protein one) induces inflammatory responses that limit proliferation of the intracellular pathogen in macrophage reservoirs [8]. Other cytokines such as IL-4 [9,10] and TGF-β [11] are associated with increased host susceptibility to the infection. In humans, polymorphism for CXCR2 as well as for Notch 3 Delta-like 1 ligand, which drives CD4 T helper 1 cell responses, contributes to susceptibility to visceral leishmaniasis and affects the outcome of the disease [12,13].

Studies of the key gene products controlling infection are important for developing interventions aimed at stimulating Th1-type responses and enhancing resistance to leishmania infection. Optimal activation of anti-leishmanial Th1 responses requires costimulatory signals triggered by the interaction of surface molecules on T cells and antigen-presenting cells. The activation of signal-transducing receptor pathways promoted by the interaction between CD40 ligand-CD40 and CD28-B7 in immunological synapses can stimulate Th1 type responses and enhance resistance to various forms of experimental leishmaniasis [14].

In the present study we investigated the role of a third class of costimulatory receptors represented by CD43 (leukosialin), which is involved in the induction of Th1 responses in other models such as autoimmune encephalomyelitis [15] and diabetes [16]. CD43 is a large sialoglycoprotein highly expressed by T cells; it is abundant on the T cell surface, and interacts with the T cell receptor to initiate signaling events during T cell priming [17]. CD43 signals potentiate the expression of IFN-γ by T cells during TcR activation of naïve cells, and the CD43 signaling pathway induces the expression of IFN-γ by effector CD4^+^ T cells and to a lesser extent CD8^+^ T cells [18].

Synergism between the CD43 and TcR signaling pathways promotes increased transcription of T-bet genes in CD4^+^ T cells and inhibits the transcription of GATA-3 genes in both CD4^+^ and CD8^+^ T cells, a commitment profile characteristic of IFN‐γ‐producing type 1 T cells [18]. Beside its dynamic role in progression of the T cell differentiation program, CD43 plays a positive role in T cell homing from lymphoid organs to peripheral tissues [16,19,20]. In the experimental meningitis model induced by lymphocytic choriomeningitis virus (LCMV), infection of CD43^−/−^ mice led to increased morbidity associated with decreased trafficking of virus-specific CD8^+^ T cells to tissues such as the brain [21]. Other studies using anti-CD43 antibody to block T cell migration to pancreatic islets in non-obese diabetic mice have highlighted the role of CD43 in the costimulation and trafficking of T cells, which can prevent autoimmune diseases such as insulin-dependent diabetes mellitus [16].

Given the positive regulatory role of CD43 in the induction of IFN‐γ‐dependent T cell responses and the homing properties of T cells to peripheral non-lymphoid organs where most interactions with pathogens take place, we sought to characterize the importance of CD43 in an experimental model of visceral leishmaniasis. In this study we evaluated the potential role of CD43 in visceral leishmaniasis using C57BL/6 wild type mice and CD43 knock-out derivatives (CD43^−/−^) on the same C57BL/6 genetic background.

## Methods

### Ethics statement

All mouse studies followed the guidelines set by the National Institutes of Health, USA. The study was approved by the Research Ethics Committee of Federal University of Rio de Janeiro, (protocol IMPPG040-07/16). Protocols for animal were approved by the Institutional Ethical Committees in accordance with international guidelines. All animal experimentation was performed in accordance with the terms of the Brazilian guidelines for animal welfare regulations.

### Animals and infection

C57BL/6 wild type control mice and C57BL/6 CD43^−/−^ knock-out (CD43^−/−^) originated from breeding colonies kindly donated by Professor Anne Sperling (University of Chicago, USA) were maintained in our animal facilities (UFRJ). Experimental infection was performed by inoculating 4–8 week-old female C57BL/6 CD43^+/+^and CD43^−/−^mice intravenously with 5 × 10^7^*Leishmania (L.) infantum chagasi* amastigotes (IOC-L 3324) obtained from infected hamster spleens.

### Intrahepatic parasite load and delayed-type hypersensitivity (DTH) assay

Thirty days after infection, mice were euthanized and the liver parasite load was evaluated in Giemsa-stained smears and expressed in LDU values (*Leishman Donovan* units of Stauber = number of amastigotes per 1000 liver cell nuclei/mg of liver weight). The DTH against *L. (L.) donovani* lysate was measured in the footpads at 28 days post-infection by intradermally injection in the right front footpad with 10^7^ freeze-thawed stationary phase *Leishmania (L.) infantum chagasi* (L579 Fiocruz) promastigotes in 100 μl sterile saline solution. Footpad thicknesses were measured as described at 0, 24 and 48 h after injection, and the values of the saline control in the contra-lateral footpad were subtracted from the reaction against the *Leishmania* antigen.

### Flow cytometry

For T cell phenotyping experiments, spleens were removed from infected and control animals. The organs were minced, washed and resuspended in PBS-FCS 5% for subsequent evaluation of cellularity, which was followed by triple or quadruple immunofluorescence staining. Cells were then fixed and analyzed by flow cytometry in a FACSCalibur flow cytometer. Analyses were done after recording 25,000–50,000 events for each sample, using a CELLQuest software (Becton Dickinson). Lymphocytes were gated based on forward and side scatter parameters, so as to avoid larger leukocytes such as macrophages and granulocytes. For determination of the cytokine mRNA transcripts expressed on T cells, CD4^+^ and CD8^+^ T cell subsets was obtained based on the expression of the CD43 marker by FACS cell sorting using anti CD3-APC, anti CD4-PERCP, anti CD8-PE and anti-CD43-FITC. After FACS cell sorting, the total RNA from isolated T cells was extracted using TRIzol (Invitrogen, Life Technologies) and reverse-transcribed to cDNA with SuperScript TM III Reverse Transcriptase (Invitrogen, Life Technologies).

### Quantification of mouse cytokine mRNA transcripts

Real-time PCR was performed with the ABI Prism 7900HT Fast Real-Time PCR System instrument (Applied Biosystems) using the qPCR SYBR Green Core Kit (Eurogentec) according to the manufacturer’s instructions. The amplification program included an initial denaturation step at 95°C for 10 min, followed by denaturation at 95°C for 15 s, and annealing and extension at 60°C for 1 min, for 45 cycles. SYBR Primers used to amplify specific gene products from murine cDNA were IFN-γ sense, 5′-cggcacagtcattgaaagcc-3′; IFN-γ antisense, 5′-tgtcaccatccttttgccagt-3′; TNF-α sense, 5′-ttctatggcccagaccctca-3′; TNF-α antisense, 5′-gtggtttgctacgacgtggg-3′; TGF-β sense, 5′-accgcaacaacgccatctat-3′; TGF-β antisense, 5′-tgcttcccgaatgtctgacg-3′; IL-17 sense, 5′- tctttaactcccttggcgca-3′; IL-17 antisense, 5′-ttcattgcggtggagagtcc-3′; IL-10 sense, 5′-tgaattccctgggtgagaagc-3′; IL-10 antisense, 5′-acaggggagaaatcgatgacag-3′; GAPDH sense, 5′-tgcaccaccaactgcttagc-3′; GAPDH antisense, 5′-ggcatggactgtggtcatgag-3′. Green fluorescence was measured after each extension step, and the specificity of amplification was evaluated by melting curve analysis. The relative gene expression levels were calculated using the comparative *C*t method (according to Applied Biosystems), where *C*t represents the threshold cycle. Every sample was run in three parallel reactions.

### Anti-*Leishmania (L.) infantum chagasi* ELISA

Isotype specific serum antibody responses were monitored by an enzyme-linked immunosorbent assay (ELISA) using the freeze and thawed lysate of stationary phase promastigotes of *Leishmania (L.) infantum chagasi* (L579 Fiocruz) as antigen. Whole parasite antigens were diluted to 2 μg/ml in PBS buffer (pH 7.0), and separately added at 100 μl/well to 96 well plate. After overnight incubation at 4°C, the plates were washed three times using PBS containing 0.05% (vol/vol) Tween 20 (Sigma, Gillingham, UK). Serial two-fold 1:100 to 1:800 dilutions of serum samples obtained from infected mice at 30 days post-infection (DPI) and normal mice as control diluted in PBS containing 0.05% Tween were added to the plates and incubated at 37°C for 1 hr. Following three washes with PBS containing 0.05% Tween, 1:5,000 dilution of peroxidase-labelled each goat anti-mouse Ig isotypes (IgG1,IgG2a and IgG2b) (Jackson ImmunoResearch, West Grove, USA) prepared in PBS containing 0.05% Tween was added at 100 μl/well and the plates incubated at 37°C for 1 hr. Following six washes with PBS containing 0.05% Tween, the reaction was developed with 50 mM phosphate/citrate buffer (pH 5.0) containing 2 mM *o*-phenylenediamine HCl and 0.007% (vol/vol) H_2_O_2_ (Sigma, UK), and interrupted with the addition of 2 M H_2_SO_4_ (50 μl/well). The ELISA plates were read at 490 nm (Spectra Max 190, Molecular Devices, Sunnyvale, USA).

### T Cell activation and Cytokine assays

For restimulation assay, splenocytes (1 × 10^6^/0,5 mL) obtained from control or infected mice at day 30 DPI were cultured in 48 well at 37°C and 5% CO2 in complete RPMI medium, in the presence or not of 10^6^ freeze-thawed stationary phase *Leishmania (L.) infantum chagasi* (L579 Fiocruz) promastigotes. After 3 days of *in vitro* stimulation, supernatants were collected and cytokine levels (IFN-γ, TNF-α, IL-10 and TGF-β) were assayed by ELISA utilizing purified and biotinylated Abs (R&D Systems), biotin-conjugated streptavidin-alkaline phosphatase (BD Pharminge) and developed with ELISA Develpment Kit from R&D System according to the manufacturer’s instructions. Plates were read at 405 nm and values are presented as pg cytokine/mL (mean ± SE).

### Statistical analysis

Statistical analyses were performed with GraphPad Prism 4 software, using one-way ANOVA and Turkey test. Results were expressed as mean ± standard error (S.E.), Differences between control and treated group were considered statistically significant when *P ≤* 0.05.

## Results

### Expression of leukosialin (CD43) is critical for resistance to visceral leishmaniasis

Visceral leishmaniasis is characterized by multiple organ infections that promote systemic immune responses. At the outset of infection the liver contains infected tissues constituting important reservoirs of parasites that are the target of anti-leishmania T cell protective immune responses [22]. We examined the relevance of CD43-mediated IFN-γ responses in antileishmanial immunity, and their role in inhibiting intrahepatic development of the parasite. To this end, we used a murine model of VL in which infection of C57BL/6 mice with *Leishmania (L.) infantum chagasi* amastigotes gives rise to a higher parasite load in the liver than in other organs such as the spleen and bone marrow during the first weeks, after which it is controlled by the host immune response.

**Figure 1:**
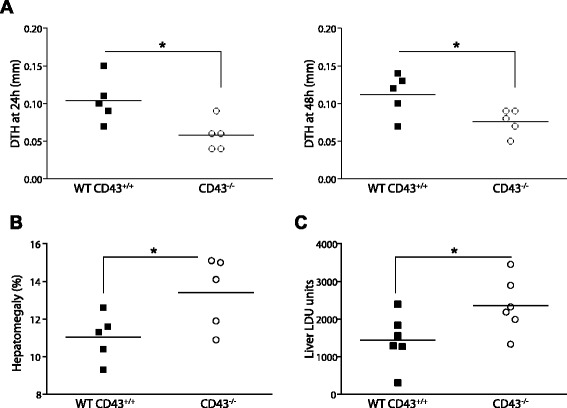
**CD43 deficient mice are susceptible to visceral leishmaniasis.** CD43^+/+^ and CD43^−/−^ mice on a C57BL/6 background were intravenously injected with 5 × 10 ^7^ amastigotes of *Leishmania (L.) infantum chagasi,* and 30 days after infection the clinical signs of disease were examined. **(A)** Infection in CD43-deficient mice results in an impaired DTH response. The vertical axis in the histogram represents the individual values of the thickness of the skin reaction in mm 24 h and 48 h after intradermal injection of 10^7^ freeze–thawed stationary phase promastigotes at 30 DPI*.* Controls with saline were done by contra-lateral injection and their values were subtracted from the reaction promoted by Leishmania antigen. **(B)** Liver/body relative weights are increased in infected CD43-deficient mice*.* The body weight was measured in grams at day 30 and the liver/body relative weights (grams of organ weight × 100 / grams of body weight) were determined in CD43^+/+^ and CD43^−/−^ mice. **(C)** Increased parasite burden in the liver of CD43-deficient mice. The vertical axis in the histogram represents the average liver parasite load in Leishman-Donovan units of Stauber (LDU = number of amastigotes / 1000 cell nuclei × organ weight in mg) obtained at 30 DPI. Data are means ± SE and represent the results of three independent experiments performed with 5–6 mice per treatment. Differences between groups are significant *(*p* < 0.05).

We observed a lower DTH response to the promastigote leishmania antigen in CD43^−/−^ mice on a C57BL/6 background than in wild type C57BL/6 mice after infection with *Leishmania (L.) infantum chagasi* (Figure 1A). These data indicate that cellular immunity against visceral infection of leishmania parasite is impaired in knockout mice, raising the possibility that CD43 is required for optimal development of host resistance to VL. We next examined the signs of acute infection in the liver. Following intravenous infection with amastigote forms of *Leishmania (L.) infantum chagasi* we observed that the increased susceptibility of CD43-deficient mice was correlated with greater increases in the liver/body weight ratio (Figure 1B) and intrahepatic parasite burden at 30 days post-infection in CD43^−/−^ mice than in the wild-type control mice (Figure 1C).

### The increased susceptibility of CD43-deficient mice infected with *Leishmania (L.) infantum chagasi* is associated with a switch in the humoral response and diminished levels of IFN-γ

**Figure 2:**
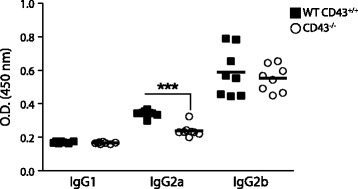
**IgG profile of serum antibodies to** ***Leishmania*** *(* ***L.) infantum chagasi*** **of CD43-deficient mice.** Sera of CD43^+/+^ and CD43^−/−^ mice infected with 5 × 10^7^ amastigote forms of *Leishmania (L.) infantum chagasi* were collected at 30 DPI and the absorbance values of IgG antibody isotypes (IgG1, IgG2a and IgG2b) were determined by ELISA using *Leishmania (L.) infantum chagasi* promastigote lysates. Bars show levels of IgG1, IgG2a and IgG2b antibodies as the individual absorbancy values of 1/100 diluted sera. Asterisks indicate significant differences between groups ***(*p* < 0.001). Data represent the individual results for each group of mice of two independent experiments.

Resistance to visceral infection by Leishmania species is associated with the IgG isotype profile [23]. Hence, we measured specific IgG antibody levels in mouse serum to determine whether the impaired resistance of CD43-deficient mice is associated with a switch in the IgG subtype profile. Serum samples were collected at day 30 post-infection and IgG titers were determined by ELISA. We found that levels of IgG2a parasite-specific antibodies in infected CD43^−/−^ mice were significantly lower than in infected wild-type mice (Figure 2). On the other hand, the IgG1 and IgG2b antibodies themselves, from both groups, did not differ significantly in their reactivity to lysates (Figure 2).

**Figure 3:**
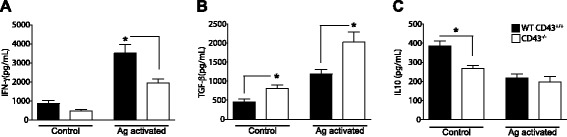
**Splenocytes from CD43-deficient mice produce low levels of IFN-γ.** CD43^+/+^ and CD43^−/−^ mice were infected intravenously with 5 × 10^7^ amastigotes of *Leishmania (L.) infantum chagasi,* and 30 days after infection splenocytes were isolated and assayed for cytokine production. After 3 days of *in vitro* culture with freeze and thawed lysates of *Leishmania (L.) infantum chagasi* promastigotes, as described in the Methods sections, splenocyte supernatants were harvested for determination of **(A)** IFN-γ, **(B)** TGF-β and **(C)** IL-10 by ELISA. The *y*-axis represents the levels of cytokines, detected by specific ELISA assays, expressed in ng/ml. Asterisks represent statistical significance between groups (p < 0.05). All experiments were repeated at least 3 times.

A major factor that is believed to contribute to healing in leishmaniasis is the development of a strong IFN-γ response, which induces IgG2a production [23]. We therefore compared the cytokine responses to infection with *Leishmania (L.) infantum chagasi* parasites by analyzing the cytokines in supernatants of splenocytes isolated from infected wild-type and knockout mice stimulated with whole parasite antigens for 3 days. Splenocytes obtained from infected wild-type mice at 30 DPI when stimulated with parasite antigen mounted a typical protective pro-inflammatory response characterized by high levels of IFN-γ and low levels of TGF-β (Figure 3A, B). In contrast, the splenocytes from infected CD43^−/−^ mice produced reduced levels of IFN-γ and high levels of TGF-β upon stimulation with parasite antigens (Figure 3A, B). This clearly demonstrates that CD43-deficient mice have impaired immune response generation after infection with *Leishmania (L.) infantum chagasi*. In fact, splenocytes from CD43^−/−^ mice spontaneously secreted TGF-β as seen in the unstimulated controls (Figure 3B) and did not decrease the production of IL-10 upon stimulation with parasite antigens as compared to wild-type cells (Figure 2C). We did not detect significant levels of TNF-α in the culture supernatants (Data not shown).

### The majority of intrahepatic CD4^+^ and CD8^+^ T cells with pro-inflammatory phenotypes express CD43 in visceral leishmaniasis

**Figure 4:**
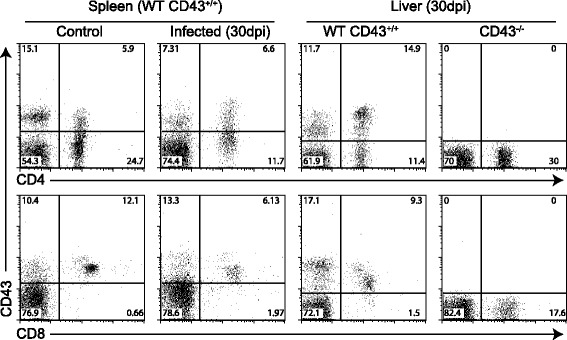
**Expression of CD43** ^**+**^ **T cells defines major intrahepatic CD4** ^**+**^ **and CD8** ^**+**^ **T cell subsets during visceral leishmaniasis.** Following infection of CD43^+/+^ and CD43^−/−^ mice with 5 × 10^7^amastigotes of *Leishmania (L.) infantum chagasi,* spleens and livers were collected at 30 DPI for FACS analysis. Splenocytes and mononuclear cells purified from livers were analyzed by FACS after staining with anti-CD43-FITC, anti-CD8-APC and anti-CD4-PE. Plots represent CD43^+^CD8^+^ and CD43^+^CD4^+^ lymphocyte pools derived from five mice. The values in the corners represent mean percentages of CD43^+^ cells in the total CD4^+^ or CD8^+^ T cell populations. Non-infected mice were used as controls. These data are representative of three independent experiments, using five mice per group.

We next examined whether the increased parasite load associated with absence of CD43 influenced the tissue distribution of intrahepatic T cells. It has been demonstrated that T cell- and lymphokine-dependent mechanisms are involved in the formation of antileishmanial tissue granulomas and the acquisition of resistance to Leishmania infection [22]. Hence we performed experiments to characterize the phenotypic distribution of intrahepatic T cells based on expression of the CD43 marker. In these experiments, wild-type mice were infected with amastigote forms of *Leishmania (L.) infantum chagasi* and the presence of CD4^+^ and CD8^+^ T cells in the spleen and liver was evaluated by FACS at 30 days post-infection. In both naïve and infected mice, the CD43^+^ subset in the CD4^+^ T cell compartment was significantly smaller than the CD43^−^ population whereas in the CD8^+^ T cell compartment, the majority of cells expressed CD43 (Figure 4). A striking difference became apparent when we examined the expression of CD43 in the intrahepatic CD4^+^ T cell population during infection, as our data indicated that the majority of the CD4^+^ T cells, like the CD8^+^ T cells, were CD43^+^ (Figure 4).

**Figure 5:**
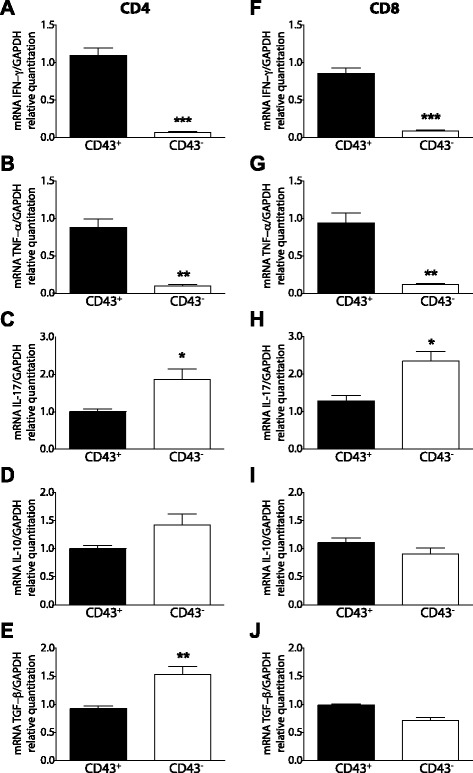
**Intrahepatic CD43** ^**+**^ **CD4** ^**+**^ **and CD43** ^**+**^ **CD4** ^**+**^ **T cells exibit a pro-inflammatory phenotype in visceral leishmaniasis.** Livers were removed from CD43^+/+^ wild-type mice infected with 5 × 10^7^ amastigotes of *Leishmania (L.) infantum chagasi* at 30 DPI and CD43^+^CD4^+^ and CD43^+^CD8^+^ T cell subsets were purified by flow cytometry. To measure cytokine transcripts, total mRNA from highly purified (>98%) T cell subsets (as indicated) were processed for RT-PCR. The results represent **(A,F)** IFN-γ transcripts, **(B,G)** TNF-α transcripts, **(C,H)** IL-17 transcripts, **(D,I)** IL-10 transcripts, and **(E,J)** TGF-β transcripts, standardized with the housekeeping gene GAPDH, calculated as 2^-ΔCt^ and presented as mean values ± SE. All experiments were performed in triplicate and the data shown are representatives of three independent experiments using five mice per group. Indicated differences between groups are significant *(p<0.05), **(p<0.01), ***(p<0.001).

Since the CD43 signaling pathway is involved in the T-bet-dependent expression of IFN-γ by type-1 T cells, we investigated the cytokine profiles of the T cell subsets based on the expression of the CD43 marker. Immunosuppression of antigen-specific immune responses due to impairment of DC activation, and CD4^+^ and CD8^+^ T cell exhaustion has been demonstrated in visceral leishmaniasis [24-26]. Indeed we found in our model that splenic T cells obtained from wild-type infected mice at 30 DPI secreted neither IFN-γ nor TNF-α upon polyclonal stimulation with an anti-CD3 stimulus (Additional file 1: Figure S1). Therefore, in order to assess the polarization profiles of the intrahepatic T cell subsets during infection we measured changes in the levels of cytokine transcripts. Total RNA was isolated from CD4^+^ and CD8^+^ T cells sorted by FACS based on the expression of the CD43 marker and purified from the livers of wild-type mice at 30 days post-infection. Real-time RT-PCR analysis revealed a significantly higher level of induction of the pro-inflammatory cytokines IFN-γ and TNF-α in the CD43^+^ subset than in the CD43^−^ population (Figure 5). At the same time we detected a greater reduction of IL-17 and the regulatory TGF-β and IL-10 cytokines in the CD43^+^ T cell subsets, suggesting a role of CD43^+^ T cells in inflammatory responses during infection (Figure 5).

## Discussion

The purpose of this study was to elucidate the role of CD43 in disease progression and immune responses in mice infected with *Leishmania (L.) infantum chagasi.* CD43 (leukosialin, sialophorin) is a large sialoglycoprotein that is abundantly expressed by cells of hemopoietic origin, including both CD4^+^ and CD8^+^ T cells [17]. Our experimental data clearly support a role for CD43 in the development of resistance to infection with *Leishmania (L.) infantum chagasi,* as our findings indicated that CD43^−/−^ mice were defective in their DTH response to leishmanial antigens and had a higher hepatic parasite load.

It has been show in other systems that CD43 is able to affect the polarization of the Th immune response [15,16,18,21]. In the *M. tuberculosis* infection model, CD43 plays a role in the uptake of *M. tuberculosis* by macrophages and in type-1 immune responses [27,28]. Some mechanisms by which CD43 controls the intracellular growth of *M. tuberculosis* have been described. These include the induction of apoptosis and the production of reactive oxygen intermediates (ROI) and RNI, which are enhanced by cytokines such as IFN-γ and TNF-α [29].

Although other studies have demonstrated a role of the CD43 signaling pathway in the innate defense of macrophages by increasing the oxidative burden during tuberculosis infection, we have ruled out this possibility in the case of *Leishmania* infection since macrophages derived from the bone marrow of CD43^+^ and CD43^−^ mice were equally effective in eliminating *Leishmania* promastigotes (Additional file 2: Figure S2). However, our results show that protective immune responses elicited via CD43 are required for the increased expression of IFN-γ, which is associated with protective immune responses in this model and other murine models of infection with *L*eishmania parasites [30].

We further showed that the low levels of IFN-γ in CD43-deficient mice infected with *Leishmania (L.) infantum chagasi* were accompanied by increased production of the anti-inflammatory cytokine TGF-β upon antigenic stimulation, so resulting in a decreased ratio of IFN-γ/TGF-β compared with wild-type infected mice. This alteration could lead to macrophage dysfunction *in vivo* as it has been shown that increased levels of TGF-β promote parasite replication inside macrophages [11]. Beside of its effect on macrophage activation, IFN-γ stimulates B lymphocytes to produce IgG2a, which plays a critical role in resistance to leishmaniasis by activating macrophage defenses through increasing parasite uptake and anti-parasite activity [23].

It is of interest that infection of CD43-deficient mice results in reduced levels of IgG2a implying that the type-1 response to infection is compromised. Our findings pointed to a critical role of the CD43 on the acquisition of host protective responses, as we observed striking differences in the liver parasite burdens under infection with *Leishmania (L.) infantum chagasi* indicating a less efficient control of the parasite replication in CD43-deficient mice during infection. It has been shown that besides its role as a regulator of immunity, the CD43 signaling pathway participates in the migration and tissue distribution of circulating leukocytes to secondary lymphoid organs and peripheral tissues [16,19,20].

We have also observed an altered distribution of CD43^+^ T cells in the periphery, as experimental infection of wild-type mice with *Leishmania (L.) infantum chagasi* yielded an increased proportion of intrahepatic CD43^+^ T cells, in both CD4^+^ and CD8^+^ subsets, with an effector profile based on the expression of the major pro-inflammatory cytokines associated with hepatic control of parasite load during the acute phase of infection [22]. Our results indicated that the weakened control of the intrahepatic parasite load was correlated with loss of the majority of CD43^+^ CD4^+^ and CD8^+^ T cells exhibiting pro-inflammatory cytokine profiles. In fact, in visceral leishmaniasis, the acquisition of intrahepatic resistance to the parasite is a consequence of the tissue expression of a protective T cell response that promotes the attraction of competent cells to potentiate the anti-microbial activity of infected Kupffer cells by inducing the production of reactive oxygen and nitrogen intermediates as well as the pro-inflammatory cytokines IFN-γ and TNF-α, which play a critical role in liver granuloma formation and control of parasite colonization [22].

## Conclusions

Together our data define, for the first time, a role for CD43 in the hepatic protective immune response against parasite reservoirs in visceral leishmaniasis. We observed that CD43^−/−^ mice were more susceptible to the disease, which implies that sialoprotein participates in disease resolution and elimination of the parasite. More specifically we demonstrated that loss of CD43 signaling pathway resulted in a modulation of the cytokine balance, with a significant decrease in the IFN-γ type 1 response, which is essential for elimination of the parasite. A successful immune response is also correlated with the diminished production of the anti-inflammatory cytokine, TGF-β, which promotes susceptibility to the disease. Understanding how host protective immune responses can affect the intracellular growth of Leishmania parasites could lead to the development of novel therapeutic or preventative measures against this devastating pathogen.

## Additional files

Additional file 1: Figure S1. Flow cytometric analysis of *intracellular* IFN-γ in T cells from visceral leishmaniasis. Spleens from CD43^+/+^ wild-type mice were collected on day 30 after infection with 5 x 10^7^amastigotes of *L.(L) infantum chagasi* to assay intracellular expression for IFN-γ. Splenocytes were stimulated with plate-bound anti-CD3 (5 μg/mL), in the presence of GolgiPlug (brefeldin A) for 6 h at 37^o^C in 5% CO_2_. Samples were stained with CD3-FITC, CD4-Cy-Chrome **(A)** and CD8-APC **(** **B** **)** and then intracellularly with IFN-γ PE as described in Methods. Results are representative of duplicate cultures from three different experiments. Additional file 2: Figure S2. Bone marrow-derived macrophages from CD43^-/-^ and wild-type cells have similar levels of infectivity with *Leishmania (L.) chagasi* promastigotes. Bone marrow-derived macrophages generated from CD43^+/+^ and CD43^-/-^ mice were infected with amastigotes at a 10:1 ratio of parasites: host cells. At 1 hour after infection, cells were washed to determine the % infection, and the cultures were incubated for a further 72 hours to measure intracellular growth of the amastigote forms. The number of intracellular parasites per 100 cell nuclei was determined after citospinning the cells onto glass slides, followed by fixation and Giemsa staining. Data represent the mean ± SEM of triplicate assays and are representative of two independent experiments.
