# Supplementary figures and images for: Expression of leukosialin (CD43) defines a major intrahepatic T cell subset associated with protective responses in visceral leishmaniasis
Source: Parasit Vectors. 2015 Feb 19;8:111. doi: 10.1186/s13071-015-0721-9 (PMC4340829; doi:10.1186/s13071-015-0721-9)

Figure S1

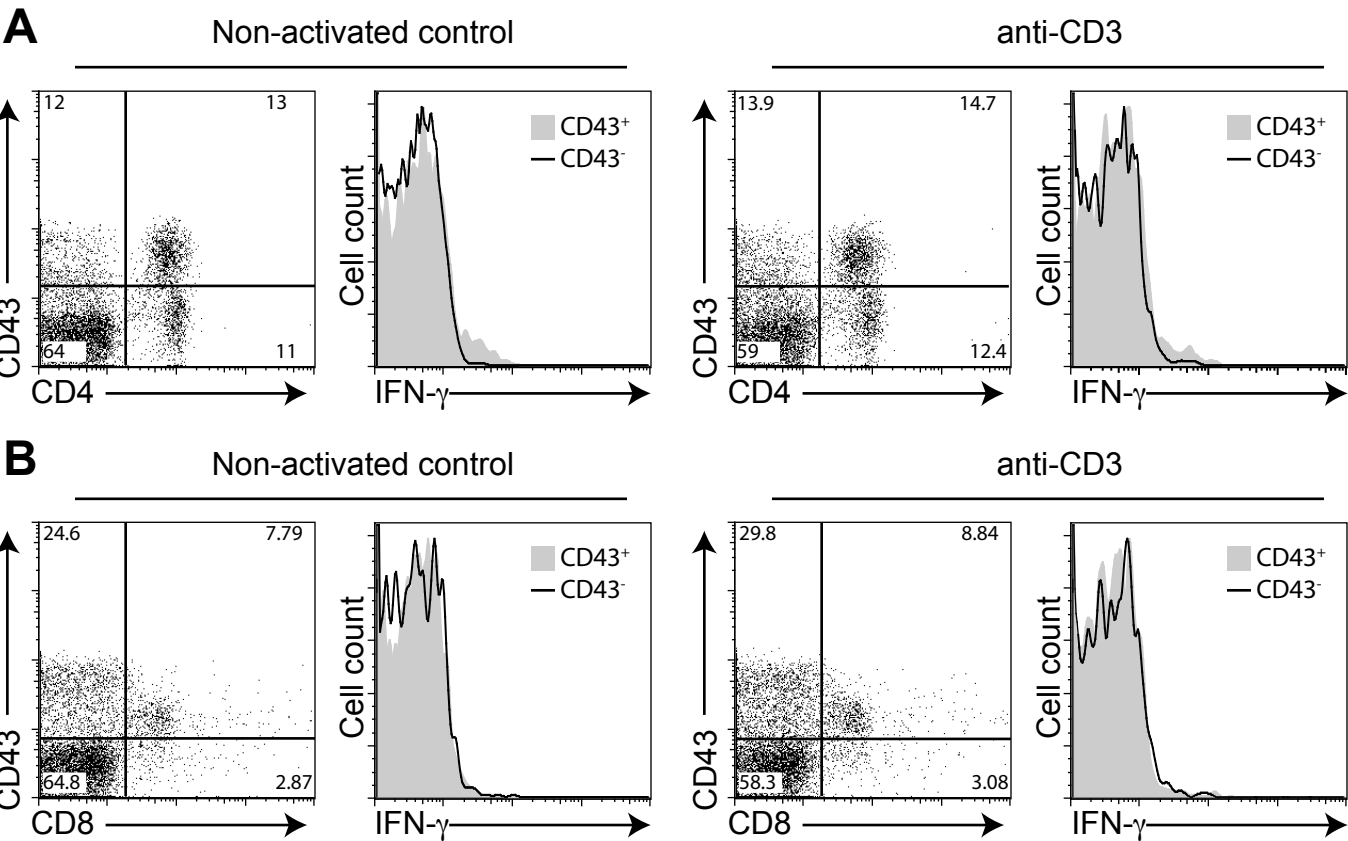

Supplement: Additional file 1: Figure S1. — Flow cytometric analysis of intracellular IFN-γ in T cells from visceral leishmaniasis. Spleens from CD43+/+ wild-type mice were collected on day 30 after infection with 5 x 10^7^amastigotes of L.(L) infantum chagasi to assay intracellular expression for IFN-γ. Splenocytes were stimulated with plate-bound anti-CD3 (5 μg/mL), in the presence of GolgiPlug (brefeldin A) for 6 h at 37oC in 5% CO2. Samples were stained with CD3-FITC, CD4-Cy-Chrome (A) and CD8-APC ( B ) and then intracellularly with IFN-γ PE as described in Methods. Results are representative of duplicate cultures from three different experiments. [file 13071_2015_721_MOESM1_ESM.pdf]

Figure S2

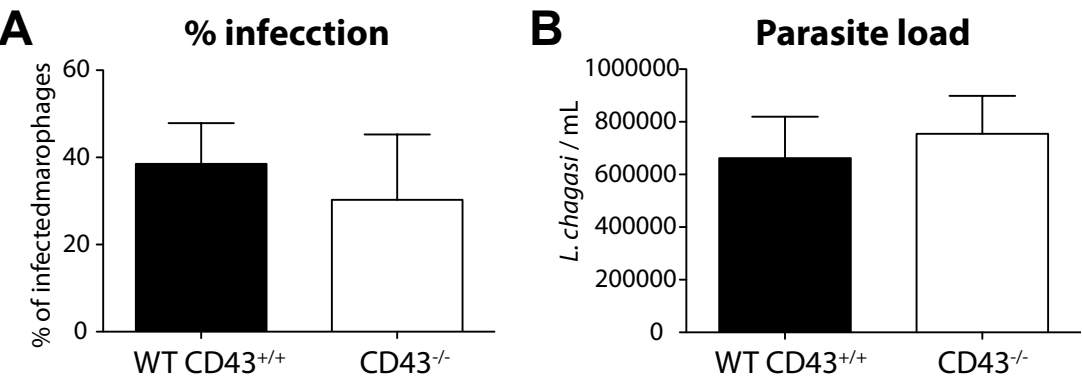

Supplement: Additional file 2: Figure S2. — Bone marrow-derived macrophages from CD43-/- and wild-type cells have similar levels of infectivity with Leishmania (L.) chagasi promastigotes. Bone marrow-derived macrophages generated from CD43+/+ and CD43-/- mice were infected with amastigotes at a 10:1 ratio of parasites: host cells. At 1 hour after infection, cells were washed to determine the % infection, and the cultures were incubated for a further 72 hours to measure intracellular growth of the amastigote forms. The number of intracellular parasites per 100 cell nuclei was determined after citospinning the cells onto glass slides, followed by fixation and Giemsa staining. Data represent the mean ± SEM of triplicate assays and are representative of two independent experiments. [file 13071_2015_721_MOESM2_ESM.pdf]
